# Oncolytic paramyxoviruses-induced autophagy; a prudent weapon for cancer therapy

**DOI:** 10.1186/s12929-019-0542-9

**Published:** 2019-06-19

**Authors:** Mohsen Keshavarz, Farid Solaymani-Mohammadi, Seyed Mohammad Miri, Amir Ghaemi

**Affiliations:** 1 The Persian Gulf Tropical Medicine Research Center, The Persian Gulf Biomedical Sciences Research Institute, Bushehr University of Medical Sciences, Bushehr, Iran; 2 Department of Virology, School of Medicine, Iran University of Medical Sciences, Tehran, Iran; 3 Department of Virology, School of Public Health, Tehran University of Medical Sciences, Tehran, Iran; 4 Department of Chemistry, Sharif University of Technology, Tehran, Iran; 5 Department of Virology, Pasteur Institute of Iran, P.O.Box: 1316943551, Tehran, Iran

**Keywords:** Oncolytic virotherapy, Paramyxovirus, Autophagy

## Abstract

Oncolytic virotherapy has currently emerged as a promising approach upon which scientists have been able to induce tumor-specific cell death in a broad spectrum of malignancies. Paramyxoviruses represent intrinsic oncolytic capability, which makes them excellent candidates to be widely used in oncolytic virotherapy. The mechanisms through which these viruses destroy the cancerous cells involve triggering the autophagic machinery and apoptosis in target cells. Interestingly, oncolytic paramyxoviruses have been found to induce autophagy and lead to tumor cells death rather than their survival. Indeed, the induction of autophagy has been revealed to enhance the immunogenicity of tumor cells via the release of damage-associated molecular patterns (DAMPs) and the activation of autophagy-related immunogenic cell death (ICD). Subsequent cross-presentation of tumor-associated antigens (TAA) through the MHC-I complex to CD8+ T cells results in the productive priming of the tumor-specific immune response. In this review, we first briefly discuss autophagy and explain the process of viral xenophagy. Finally, we focus on the interactions between virus and autophagy proteins, elaborating on the global preclinical studies on oncolytic paramyxoviruses.

## Background

Cancer poses a significant threat to the global health and is known as the second most common cause of death after cardiovascular diseases, owing to its high recurrence and mortality rates which have been estimated to be nearly 9.6 million deaths in 2018 [1].

Chemotherapy is still the dominant therapeutic approach for the majority of human invasive malignancies. However, traditional direct administration of therapeutic drugs to the patients is no longer applicable due to their intrinsic limitations including undesirable side effects, unfavorable pharmacokinetics, and poor bio-distribution. Thus, development of an efficient weapon with novel mechanisms of action (MOA), minimal toxicity, and significant specificity is urgently required to effectively destroy tumors even after metastasis [2].

Virotherapy has been defined as an emerging therapeutic strategy against cancer. Oncolytic viruses (OVs) in both forms of naturally occurring and genetically engineered viruses can selectively replicate in and kill malignant cells without harming healthy ones [3]. Direct lysis of tumor cells [4] along with indirect induction of acquired immunity against tumor-specific antigens (TSAs) are two principal mechanisms through which OVs play their therapeutical roles. Approval of the first oncolytic virus (T-Vec, talimogene laherparepvec) by the US Food and Drug Administration (FDA) and the European Medicines Agency (EMA) for the treatment of non-resectable melanoma has provided a novel class of anti-cancer agents in the field of cancer therapeutics [5]. Regardless of several achievements and also the announcement of the first commercially available product, there are many challenges to be addressed in the use of oncolytic virotherapy for the treatment of patients suffering from cancer (e.g., high pathogenicity, antiviral immune responses, and incomplete targeting to all sites of established tumors). Therefore, development of a new generation of virus-related oncolytic therapy for overcoming these challenges is highly in demand.

The paramyxoviruses (members of the Paramyxoviridae family, order Mononegavirales) are enveloped, non-segmented negative-sense RNA viruses with 100–300 nm diameter that cause diseases among both animals and humans [6]. In-vitro studies have shown that the members of this family (Newcastle disease virus, measles, morbillivirus, and respiratory syncytial virus) could naturally replicate and disrupt different tumor cell lines [7]. Various mechanisms have been proposed for tumor tropism of oncolytic paramyxoviruses ranging from overexpression of the viral-specific receptors on cancerous cells, cell death due to tumor-specific syncytia formation, apoptosis, autophagy, and immune cell death (ICD) to cytotoxic immune effector-induced cytotoxicity [8].

Autophagy is a crucial biological process during which a double layer membrane engulfs cytosolic cargo as well as infectious agents and create autophagosomes in response to fusion with lysosomes. Although autophagy plays a fundamental role in degrading during viral infections, other viruses have evolved several evasion strategies to escape to escape from the host autophagy for their own benefit including blocking autophagosome maturation or degradation or inhibition of autophagy components [9, 10]. Ultimately, hydrolytic enzymes of lysosomes degrade and recycle autophagosomal contents. Thus, autophagy is a critical process in cell growth, control of damage, nutrient starvation, and other cellular stresses such as oxidative stress and infection [11]. It also has a vital role in caspase-independent autophagic cell death through the release of damage-associated molecular patterns (DAMPs) during viral infections [12]. A growing body of evidence has revealed that by incorporation of sophisticated evolved mechanisms during infection, some viruses evade from autophagy and some others hijack autophagy components for their own replication and spread. Hence, the role of autophagy as an intracellular protection strategy in viral infections is totally complicated [13].

This review focuses on oncolytic paramyxoviruses (Newcastle disease virus, measles, Sendai and morbillivirus) and highlights the function of autophagy in the replication and infection of these viruses as well as the role of viral proteins in antagonizing or manipulation of autophagy machinery.

### Autophagy

Based on morphological appearance, cell death is categorized into three major types: apoptosis, autophagic cell death, and necrosis [14]. Autophagy is basically considered as a catabolic and highly conservative cellular process [15] which plays a cardinal role in the maintenance of cellular hemostasis via eliminating the destructed cellular organelles, exhibiting cytoprotective effects [16].

In this regard, the aggregates of proteins with the capability of selective removal of intracellular microbes play vital roles in xenophagy [17, 18]. Xenophagy initiates with the recognition of ubiquitin chains expressed on pathogens, mediated by several receptors such as p62 sequestosome protein or neighbor of breast cancer gene 1 protein (NBR1). The latter is equipped with a microtubule-associated protein light chain 3 (LC3)- interacting region (LIR) motif which enables this receptor to interact with ATG8/LC3 and facilitates the target delivery to autophagosomes [19]. In spite of being a cytosolic protein, LC3 is covalently linked to phosphatidylethanolamine, leading to its localization to autophagosomal membranes and formation of membrane vesicles [20]. A wide range of stimulants has been proposed to activate autophagy, including deprivation of nutrients, oxidative stress, endoplasmic reticulum stress, and infection with microbial agents [21]. Autophagy takes place in two distinct stages: 1) formation of phagophore or isolation membrane through various cellular membrane sources, 2) subsequent development of phagophore membrane, which leads to the formation of a double—membrane structure, namely autophagosome. These structures become matured through fusion with lysosomes and form autolysosomes [22]. Autophagy is known as a tightly regulated process. It has been demonstrated that various regulatory factors control the initiation of this phenomenon, including mTOR complex 1 (mTORC1) and AMP-activated kinase (AMPK) [23].

Two principal hallmarks have been proposed to identify genuine and functional autophagic reactions, 1) presence of cytoplasmic materials in these reactions, and 2) the peak occurrence in the presence of lysosomal degradation. More than 30 autophagy-related (ATG) genes have been identified as playing crucial roles in the executive process of autophagy. These genes take part in a number of specific steps of autophagosome biogenesis, including initiation with UNC-51-like kinase 1 (ULK1) complex, nucleation of the vesicle with Beclin-1-class III phosphatidylinositol 3-kinase complex, and elongation and fusion with microtubule-associated protein light chain 3 lipidation and sorting nexin 18 (SNX18) complex, respectively [24–28]. ATG12 and ATG8 are two ubiquitin-like systems of conjugation which belong to the autophagic machinery and have been conserved in the course of evolution. The recruitment of these proteins to the membranes of autophagosomes is observed with their formation, maturation, and development [29].

There are six proteins which belong to the family of ATG 8 proteins (also known as LC3 proteins), including LC3A, LC3B, LC3C, GABARAP, GABARAPL1, and GATE-16. Cytosol is the place where the unlipidated form of these ubiquitin-like proteins can often be found. A number of ATG proteins which activate ubiquiting-like cascades are required to mediate the conjugation of LC3 proteins to phosphatidylethanolamine which is found on the phagophore. In summary, a glycine residue is exposed following the removal of C-terminal amino acids before the lipidation of ATG4 protease, being used in the first steps of ATP-mediated thioester linkage with a cysteine residue found in E1-like ATG7 [30].

The complex of ATG12 ∼ ATG5–ATG16L1 exhibiting functions resemble to E3 can be constituted during the second cascade in which ATG12 is involved and makes covalent E1 ATG7- and E2 ATG10-mediated conjugation with ATG5. Dimerization of ATG12 ∼ ATG5 is driven by the interaction of ATG16L1 and ATG5. The WIPI2b acts in the recruitment of complex to phagophore. Ultimately, a covalent bond can be established between LC3 and amine headgroup of PE through the interaction between ATG12 and ATG3, leading to the formation of lapidated LC3-II. LC3-II then can act in the decoration of phagophore’s outer and inner membranes [30].

While autophagy is harnessing powerful cytoprotective activity, autophagy-triggered cell death is observed in some cases, which leads to uncontrolled autophagy. LC3 interaction motif (LIM) also know as LC3 interaction region (LIR) mediate the interaction between autophagy receptors and ATG8/LC3/GABARAP, allowing for cargo recognition during selective autophagy [31].

### Oncolytic paramyxoviruses and autophagy

**Fig. 1:**
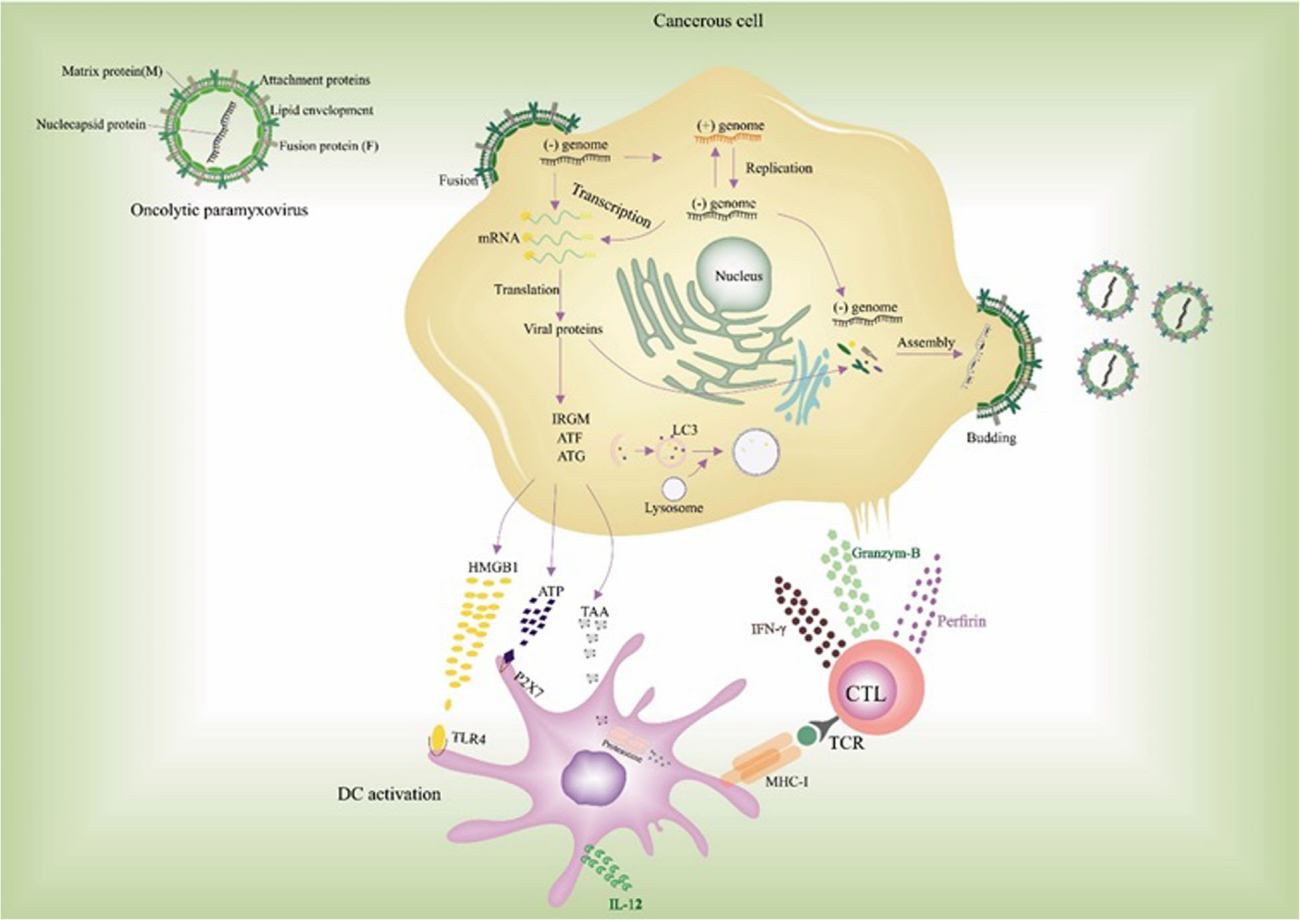
Oncolytic paramyxoviruses infect and preferentially destroy tumor cells. Following OVs replication, cancerous cells release TAA and DAMPs, both of which can be recognized by PRRs on APCs leading to the release of inflammatory cytokines. Also, mature APCs present TAAs through MHC-Ӏ to tumor-specific CD8+ T cells and thereupon, antitumor immune response leads to the lysis of tumor cells via the release of perforin, granzyme B, and IFN-gamma

Viral xenophagy (virophagy) is defined as an autophagic reaction which is employed to attack cytoplasmic virions or their components [32]. Facilitation of viral replication utilizing autophagic machinery, efficient viral replication in the presence of impaired autophagic flux, and hindered viral replication are three leading consequences of virally driven manipulation of autophagy. Indeed, a number of viruses exhibit various interactions with autophagy components regarding the host cell type [13]. For instance, while the HIV Nef protein inhibits the transition from autophagosome to autolysosome in macrophages, HIV takes advantage of autophagic machinery to replicate. This function of Nef protein helps the virus recover after proteolytic degradation. As a result, distinct viral pathogenesis is attributed to different modulatory effects of autophagy on various strains or serotypes of viruses [33]. Several lines of evidence have revealed that paramyxoviruses deploy autophagy as a way to guarantee their replication, which can be triggered by viral glycoprotein-mediated membrane fusion. For instance, Human Parainfluenza Virus 3 (HPIV3) is responsible for autophagosome maturation blockade [34–36]. Autophagosomes accumulation delineates the equilibrium between their generation rate and conversion to autolysosomes. Therefore, during HPIV3 infection, incomplete autophagy is induced through blocking autophagosome-lysosome fusion and subsequent accumulation of autophagosomes and suppression of the autophagic flux. A study by Ding et al. demonstrated that two SNARE motifs of SNAP29 can mediate the HPIV3 phosphoprotein (P)-SNAP29 interaction which interferes with the phagosome-lysosome fusion, leading to an effective production of virus particles. A competitive binding of P to SNARE motifs of SNAP29 and Stx17 was found in this study. As several lines of evidence have also indicated the pivotal role of other SNARE proteins in the regulation of autophagosome-lysosome fusion and also the recruitment of LC3 to the place where autophagosome forms, a variety of viruses can take advantage of these proteins to inhibit the autophagy maturation [37]. It has been well documented that paramyxoviruses are able to induce the syncytia creation through which infected cells fuse with their neighborhood cells and give rise to the establishment of giant multinucleated cells. The ability to produce syncytia is another enormous privilege of the paramyxoviruses which enables them to disseminate without the release of any mature virus from the infected cells [38]. In this regard, syncytia formation highly facilitates the replication of viruses without the encounter with neutralizing antibodies, which in turn enhances the potential of paramyxoviruses in providing efficient viral oncolysis [8]. Besides, immunogenic syncytia secrete plenty of syncytiosomes, presenting tumor-associated antigens (TAAs) via MHC molecules [39, 40]. There is an association between syncytia death and autophagy. It has been demonstrated that infection with several members of the family paramyxoviridae, including Measles virus (MeV), Newcastle disease virus (NDV), and Sendai virus (SeV) is followed by autophagic programmed cell death [41–43]. The approved capability of antigen donor cells in antigen cross-priming, provided by autophagy, gives rise to the development of TAA-specific or virus-specific CD8+ T cells, which in turn sheds lights on the development of novel modalities to upgrade antitumor effects mediated by oncolytic viruses. Furthermore, membrane glycoproteins of paramyxoviruses with fusion capabilities are shown to vigorously enhance the DC-mediated cross-presentation of TAAs (Fig. 1) [40, 44].

### Key oncolytic paramyxoviral proteins in autophagy

In the case of MeV, it has been demonstrated that autophagy induction occurs via three separate routes [45–47]. The first pathway is related to the CD46 involvement and is observed only within attenuated strains of MeV, which results in the induction of autophagy upon virus entry [48, 49]. The second pathway initiates in a few hours after infection and occurs along with the expression of MeV-C protein accompanied by its interaction with autophagy-regulating immunity-Related GTPase family M protein (IRGM) [47]. In the final pathway, autophagy is induced through cell-cell fusion, maintaining both the replication of viral particles and the viability of syncytia [41]. Accordingly, the relationship between MeV and autophagy exhibits a high degree of complexity and therefore, the ability of MeV to take advantage of autophagy relies on the full occurrence of this process, which leads to the efficient production of viral particles [50].

Nonetheless, with regard to the presence of autophagy receptors which act in transferring of pathogens to the autophagy machinery for degradation, the mechanism through which MeV escapes from degradation by autophagy has not been fully understood [51]. Autophagy receptors are capable of binding to intracellular pathogens or their related components, thereby targeting them toward the development of autophagosomes. LC3 interacting regions (LIR) are parts of the autophagy receptors which are capable of interaction with small Ub-like proteins (UBLs) from the ATG8/GABARAP family. These regions are linked to the lipid phosphatidylethanolamine (PE) that is profoundly present in the autophagosomal membrane. Receptors containing LIR can make a bridge between the cargo and a group of proteins that are included in membrane (Atg8/LC3/GABARAP), allowing the accessibility of the phagophore-associated autophagic machinery for cargo [52, 53]. Moreover, Nuclear Domain 10 Protein (NDP52), optineurin (OPTN), and T6BP have been elucidated to have a concomitant association with phagophores’ biogenesis. Recent reports have delineated the role of NDP52 and OPTN in autophagosomes self-maturation, leading to the formation of phagosome-lysosome fusion [54, 55]. As a result, a dual action is proposed for the proteins mentioned above during xenophagy, including (i) acting as autophagy receptors to facilitate the autophagy function against pathogens, and (ii) regulating the fusion of autophagosome to lysosome in order to mediate the degradation of entrapped pathogens. T6BP was also determined to have a similar dual function, especially during bacterial infections [56].

It has been reported that the interactions between some specific proteins of autophagy machinery with several MeV proteins result in the induction of the late wave of autophagy. Among these interactions, the alleged targeting of GOPC (Golgi-associated PDZ and coiled-coil motif-containing protein) by MeV-C does not influence the infection-induced autophagy [57, 58]. The possible role of this interaction is to limit the function of GOPC in already infected cells in order to generate perturbation during further autophagy, inducing by newly entered viruses. At least, two MeV proteins, MeV-C and MeV-N, have been found to target IRGM (Immunity-Related GTPase M) and interfere with the regulation of infection-mediated autophagy. MeV-C protein, which its overexpression alone has been demonstrated to be sufficient for the IRGM-dependent autophagy induction, is able to interact with IRGM. Investigations have revealed that IRGM could interact with a number of autophagy-associated proteins, which are engaged in the early steps of autophagy. These proteins include ATG5, ATG10, LC3C (Light Chain 3 C), SH3GLB1 (SH3 Domain Containing GRB2 Like, Endophilin B1), ULK1, and BECN1 (Beclin 1), all of which could play roles in the induction of autophagy following the expression of MeV-C protein [59, 60]. The initiation of autophagosomal membrane formation has been shown to be highly dependent on the interaction of IRGM with ULK1 and BECN1 [59]. Following infection with HCV, MeV, and several other RNA viruses, IRGM is extremely essential for the ULK-1 phosphorylation, which is a crucial process in autophagy initiation [61]. LC3C is a member of LC3 subfamily, which is also necessary for the elongation of phagophore through lipidation with phosphatidylethanolamine.

Additionally, as SH3GLB1 is one of the molecular switches with the ability to promote autophagosome formation, it can interact with UVRAG (a regulator of membrane trafficking during autophagy) and thus facilitate the last step of infection-induced autophagy [62]. A wide spectrum of conditions such as viral infections can interfere with the ER function and result in unfolding or misfolding of ER proteins, a phenomenon which is called ER stress. There is also a process known as unfolded protein response (UPR), during which ER takes advantage of a set of compatible mechanisms to prevent cell death complications. In recent years, research has shown that these pathways may be related to the autophagic response that plays a key role in cellular response to inducers of stress [63–65]. NDV is one of these viruses which can induce autophagy both in vivo and in vitro, which is beneficial for its replication [66, 67]. Two proteins of NDV, P and NP proteins were shown to induce autophagy in A549 cells. The capability of these proteins in altering the hemostasis of ER is mediated by the upregulation of ER stress marker proteins, GRP78 and GRP94 [68].

Moreover, it has been shown that the PERK and ATF6 pathways are involved in the regulation of P- and NP-mediated autophagy induction and knockdown of each interferes with NDV replication [68].

**Fig. 2:**
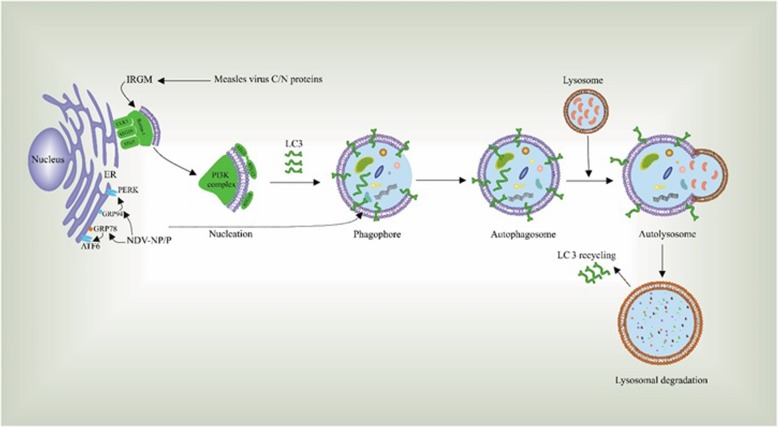
Several NDV and MeV proteins interact with autophagy-related proteins and thereby play a key role in the induction of autophagy in tumor cells. This lysosomal degradation takes place upon several stages, including nucleation, the formation of phagosomes, autophagosomes, and autolysosome

Furthermore, several studies have indicated the pivotal role of class III PI3K/Beclin-1 pathway in the NDV-induced autophagy. This pathway is also highly essential during the NP- or P-induced autophagy, as the knockdown of Beclin-1 declines the conversion of LC3 and degradation of p62 in cells transfected with NP or P proteins. Expression of these proteins activates the PERK and ATF6 pathways and leads to partially suppressed autophagic process. These data highlight the remarkable capability of PERK and ATF pathways in triggering NDV-induced autophagy. However, as the autophagy was observed in PERK- or ATF6- knockdown cells, other pathways may also play a role in the regulation of NDV-induced autophagy in a manner other than through ER stress (Fig. 2) [68].

### Newcastle disease virus (NDV)

Newcastle disease viruses belong to the Avulavirus genus, family Paramyxoviridae, and fall into three groups (velogenic, mesogenic, lentogenic) regarding their pathogenicity and virulence in the hosts [69]. Several investigations have demonstrated that the velogenic NDV (MTH68/H, Lasota, PV-701, and 73-T) could specifically infect and destroy tumor cells.

As briefly discussed above, excessive ER stress is observed in NDV-infected cancer cells, triggering autophagy via PERK/eIF2α, IRE1/JNK, or possible caspase3-mediated cleavage of eIF2α. IRE1/JNK pathway is involved in the release of beclin-1 and thereby induces the formation of autophagosomes and facilitates the LC3-II conversion. Consequently, it can enhance the autophagy through the attenuation of beclin-1 inhibition, which is mediated by the downregulation of bcl-2 [70].

A recent study reported the induction of autophagy following NDV infection in malignant cells. Meng et al. (2012) first reported that the infection of U251 cells with NDV boosts the formation of autophagosomes through the transition from LC3-I to LC3- II. They further reported that the phosphatidylinositol 3-kinase (PI3K)/Beclin-1 pathway plays an integral role in the induction of autophagy by NDV and facilitates the replication of the virus [67].

In line with previous experiments, we have determined oncolytic activity of NDV on the TC-1 cells, an E7-expressing murine tumor model. The results reported that NDV can suppress growth of tumor cells through triggering of autophagic cell death via ROS induction [71].

In addition, the induction of autophagy following NDV infection was investigated in both chicken-derived fibroblast and primary chicken embryo fibroblast (CEF). Results of the evaluation of p62/SQSTM1 degradation, LC3-II turnover, and GFP-LC3 signal revealed that the NDV infection could induce autophagy in these cells. Indeed, this virus takes advantage of autophagy for a more efficient replication [72]. In another study, pharmacologic modulation of autophagy was assessed aiming to enhance the oncolytic potential of NDV strain FMW (NDV/FMW) in drug-resistant lung cancer cells (A549 resistance to cisplatin or paclitaxel). The results indicated that the NDV/FMW induces autophagy through the modification of PI3K/Akt/mTOR/p70S6K pathway. Combination of chloroquine or rapamycin with NDV significantly promoted the oncolytic efficiency of NDV/FMW in mice bearing the drug-resistant lung cancer [73]. Research on lung cancer stem cells (CSCs) also demonstrated that the NDV-FMW induces autophagy process in these cells through the (LC3) II and P62 degradation as well as inhibition of the AKT/mTOR pathway, offering these viruses as novel and promising modalities for cancer treatment (Table 1) [74].

**Table 1:** The interactions between oncolytic paramyxoviruses and autophagy pathway

Virus .	Proteins induce autophagy	Cell/Tumor model	Autophagy/ ICD induction	Citation (s)
Newcastle disease virus	NP/P	U251	LC3-I to LC3- II conversion	Meng et al. (2012)
		CEF, DF-1	LC3-I to LC3- II conversion	Sun et al. (2014) [72]
		A549 resistant to cisplatin	PI3K/Akt/mTOR/p70S6K pathway	Jiang et al. (2014) [73]
		Lung cancer stem cells (CSCs)	Degradation of (LC3) II and P62 proteins, inhibition of the AKT/mTOR pathway	Hu et al. (2015) [74]
		GL261	Calreticulin surface exposure, the release of HMGB1 and increase in PMEL17 cancer antigen expression.	Koks et al. (2014) [79]
		A549, H1650, H460	Induction of secreted HMGB1 and HSP70/90 release.	Ye et al. (2018) [78]
Measles virus	C/N	NSCLC NSCLC	Triggering SQSTM1/p62-mediated mitophagy	Xia et al. (2014) [84]
		Hela	Induction of autophagy through cellular receptor CD46 and the scaffold protein GOPC	Richetta et al. (2013) [41]
Sendai virus	NA	NSCLC, Hela, A549	Triggering autophagy through the PI3K/Akt/mTOR/p70S6K pathway	Zhang (2015) [88] and Wang (2018) [89] et al.
		PC3	Triggering autophagy through the JNK, p38, and PI3K/beclin-1 pathway	Miao et al. (2018) [89]
		Calu-3	Induced necrosis	Zhirnov [90]
morbillivirus	NA	Vero	Vps34/beclin1 autophagic complex	Delpeut et al. (2012) [35]

*NSCLC* non-small cell lung cancer, *PC3* human prostate cancer cell line, *Calu-3* human lung cancer cell line, *NP/P* nucleocapsid protein/ phosphoprotein, *C/N* non-structural C/ nucleocapsid protein, *NA* not available

### NDV induces autophagy-mediated immunogenic cell death

Immunogenic cell death (ICD) is referred to as changes in cell surface compositions (i.e., calreticulin) which result in the secretion of soluble mediators such as ATP, high-mobility group box 1 protein (HMGB1), and Heat shock proteins (HSP) HSP70/90. Following these changes, dendritic cells (DCs) could be activated and present antigens to T cells. Once tumor cells were infected with oncolytic viruses, the release of pathogen-associated molecular patterns (PAMPs), tumor-associated antigens (TAAs), and damage-associated molecular patterns (DAMPs) could induce ICD and give rise to stronger immune response (Fig. 1) [75–77].

As mentioned above, apoptosis, autophagic cell death, necroptosis, and ER stress are among the phenomena involved in the induction of ICD following exposure to various stimuli. Preclinical experiments and clinical trials have revealed that the oncolytic NDV exhibits strong anti-tumor function. While this virus is shown to induce apoptosis, autophagy-related cell death, and necroptosis in various types of cancers, a current study on glioma cells indicated the NDV-induced Immunogenic cell death [78].

A study on glioblastoma multiforme cell lines (GL261) in xenotransplant models showed that the infection of tumor cells with NDV leads to the elevated level of cell surface calreticulin as well as the increased secretion of HMGB1 and PMEL17 tumor antigens. Following virotherapy, the results revealed a population growth among IFN-gamma^+^ CD4^+^/CD8^+^ T cells in the tumor microenvironment along with a decline in the number of myeloid-derived suppressor cells (MDSCs) [79].

A recent study (Ye et al. 2018) indicated that the oncolytic NDV mediates ICD in the lung cancer cells. Additionally, the infection of lung cancer cells with NDV could increase the levels of ATP, HSP70/90, and HMGB1 in cell supernatant. Moreover, confocal and immunoblotting analyses revealed that the expression of calreticulin is up-regulated in lung cancer cells [78]. Finally, administration of oncolytic NDV was shown to inhibit the tumor cells growth through the activation of antitumor immune responses and also the release of inflammatory cytokines (Table 1).

### Measles virus (MeV)

Measles virus belongs to the genus Morbillivirus in the family Paramyxoviridae. Measles is a highly transmissible infectious disease and is one of the most important causes of global death among young children [80]. The disease is caused by the measles virus and remains a significant cause of child mortality in developing countries [81]. Most in vitro studies and preclinical trials on oncolytic measles virus have used the attenuated vaccine Edmonston strain, which is derived from the common wild-type measles virus and is observed to have minimal side effects. Edmonston strain selectively replicates in and destroys neoplastic cells [82]. The oncoselectivity of Measles virus is chiefly based on the overexpression of the CD46 receptor on malignant cells [83]. Therefore, Measles virus is potential for the constitution of a competent, safe oncolytic group. Besides, the oncolytic virus activates the sequestosome 1(SQSTM1/p62)-mediated autophagic machinery to reduce the mitochondrion-tethered mitochondrial antiviral signaling protein (mitophagy) and then abrogates the innate immune response in nonsmall cell lung cancer (NSCLC) cells which results in enhancement of viral propagation. The results evinced that autophagy inducers could enhance the antitumor efficacy of virotherapy [84]. In another study, it was determined that autophagy contributes to the regulation of Measles virus vaccine strain Edmonston, B-induced nonapoptotic cell death, and oncolysis [85]. Moreover, it was determined that HeLa cell infection with oncolytic measles virus is associated with the autophagosome accumulation via the engagement of the CD46-Cyt-1/GOPC pathway. Investigation of the molecular pathways and autophagy kinetics by evaluation of ATG5 expression led to the discovery of two continuous waves of autophagy, the early wave and the late one. The results explained that different molecular mechanisms contribute to the autophagy induction after treatment with oncolytic measles virus vaccine strain Edmonston (Table 1) [41].

### Other members

Hemagglutinating virus of Japan envelope (HVJ-E; sendai virus) is an RNA virus, belonging to family Paramyxoviridae, genus and species Respirovirus [86]. Sendai virus, also recognized as Murine parainfluenza virus comprises the Human parainfluenza virus 3, Bovine parainfluenza virus 3, and Human parainfluenza virus 1 species [87]. It has been demonstrated that oncolytic HVJ-E induces both apoptosis and autophagy in non-small cell lung cancer cell (NSCLC). The results reported that the HVJ-E-induced autophagy is triggered and modulated by inhibition of the class I PI3K/Akt/mTOR/p70S6K pathway that negatively regulates autophagy [88]. Furthermore, it was shown that extracellular signal-regulated kinase (ERK) and the PI3K/Akt/mTOR/p70S6K pathways contribute to oncolytic HVJ-E-induced autophagy in infected HeLa cells [89]. Miao and colleagues tried to investigate the HVJ-E-mediated autophagy and apoptosis with the focus on prostate cancer (PC3) cells and also to identify the potential mechanisms that facilitate these processes. In total, HVJ-E was found to several pathways such as JNK, p38, and PI3K/beclin-1 in the studied cells in a ROS-mediated manner, leading to the occurrence of autophagy and apoptosis. These findings broaden the horizon about understanding the strategies through which HVJ-E could act as an antitumor agent and pave the way for establishing effective therapeutic modalities against cancer [90]. Another work attempted to evaluate the induction of apoptosis, necrosis, and autophagy pathways following SeV infection in Calu-3 cell lines. Viruses were shown to activate different pathways which take part in the induction of cell death. There was particular characteristics representative of necrosis including cellular swelling and failure in several processes such as chromatin DNA laddering, activation of caspase 3 and 8 pathways, and cleavage of PARP protein. There was also the activated Akt (a protein kinase with antiapoptotic activity). This study also showed that the intracellular autopghagic machinery in SeV-infected cells correlated with type of death. Specifically, there was association between suppressed apoptosis and the induction of autophagy. Therefore, SeV-infected cells lacked apoptosis while representing autophagy in a considerable level. SeV-induced necrosis was also found for the first time the studied cell line [91].

Products of IFN stimulated genes (ISG) play immunoregulatory roles and can also trigger antiviral immune responses. These products also affect the innate immune responses through manipulation and regulation of autophagy. Two strategies have been suggested: 1) playing role in the development of autophagy-based acquired antiviral immune responses or preventing viral agents from utilizing autophagy for their benefit, and 2) attacking paramount molecules responsible for antiviral immune responses which lead to a specified activation of autophagy and thereby acting as the regulators of IFN-induced responses against viruses [92].

IFN-β and ISGs are shown to be induced following paramyxoviruses infection in a transcriptional level, whose proteins can specifically interrupt several steps of the virus life cycle and hinder viral particle development. In a study by Subramanian et al. Tudor domain containing 7 (TDRD7) block the replication of SeV. This ISG is demonstrated to hamper the function of adenosine 5ˊ-monophosphate-activated protein kinase (AMPK) and thereby prevent the SeV from development of autophagy. AMPK stimulate the autophagic machinery via mTORC1 suppression and ULK1 activation [93].

Canine distemper virus (CDV) is a globally distributed pantropic morbillivirus. Morbilliviruses infect the immune cells and then spread to the epithelial cells. The viral hemagglutinin glycoprotein attaches and enters into the target cells after glycoprotein-mediated membrane fusion [94].

The role of autophagy in mouse-adapted canine distemper virus infection was evaluated and it was shown that viral glycoprotein-mediated membrane fusion correlates with enhanced autophagy and LC3 punctum formation in induced multinucleated cells, suggesting that the formation of syncytia, in turn, triggers autophagy and efficient viral propagation in virally infected cells (Table 1) [35].

## Conclusion

In the past decade, considerable progress has been made in understanding the complex interactions between autophagy and paramyxoviruses in both in vivo and in vitro experimental systems. The cancer cell selectivity and tumor cell death induction make oncolytic viruses-based therapy a promising tool for cancer therapy. Oncolytic paramyxoviruses, via positive regulation of autophagy as well as the release of damage-associated molecular patterns (DAMPs) and tumor-associated antigens (TAA) from tumor cells, potentially stimulate the innate and tumor- specific adaptive immune responses and could be designed for clinical autophagy-based, therapeutic approaches. Currently, a few clinical trials are ongoing to evaluate the effect of oncolytic virus-modulated autophagy in the treatment of human cancers.

In this review, we have focused primarily on distinct paramyxoviruses that interact with the autophagy pathway. Pre-clinical studies performed on oncolytic paramyxoviruses have demonstrated that the PI3K/Akt/mTOR/p70S6K pathway plays a critical role in autophagy and immunogenic cell death (ICD) in cancerous models. Thus, precise identification of the ways through which oncolytic paramyxoviruses induce autophagy and ICD can pave the way for improvement of their anti-cancer therapeutic potential and provided a basis for the development of a potential combinational therapeutic approaches using oncolytic paramyxoviruses and autophagy modulators.

## Data Availability

Not applicable.

## Abbreviations

AMPK: AMP-activated kinase
ATG: Autophagy-related genes
BECN1: Beclin 1
CDV: Canine distemper virus
CEF: Chicken embryo fibroblast
CSCs: Cancer stem cells
DAMPs: Damage-associated molecular patterns
EMA: European Medicines Agency
FDA: Food and Drug Administration
GOPC: Golgi-associated PDZ and coiled-coil motif-containing protein
HMGB1: High-mobility group box 1 protein
HSP: Heat shock proteins
HVJ-E: Hemagglutinating virus of Japan envelope
ICD: Immunogenic cell death
IRGM: Immunity-Related GTPase family M protein
LC3: Light chain 3
LIR: LC3-interacting region
MDSCs: Myeloid-derived suppressor cells
MeV: Measles virus
MOA: Mechanisms of action
mTORC1: Mammalian target of rapamycin complex 1
NBR1: Neighbor of breast cancer gene 1 protein
NDP52: Nuclear Domain 10 Protein
NDV: Newcastle disease virus
NSCLC: Nonsmall cell lung cancer
OPTN: Optineurin
OV: Oncolytic virus
PAMPs: Pathogen-associated molecular patterns
PI3K: Phosphatidylinositol 3-kinase
SeV: Sendai virus
SH3GLB1: SH3 Domain Containing GRB2 Like, Endophilin B1
SQSTM1: Sequestosome 1
TAA: Tumor-associated antigens
TSA: Tumor-specific antigen
ULK1: Unc-51 Like Autophagy Activating Kinase 1
