# Correction to: Oncolytic paramyxoviruses-induced autophagy; a prudent weapon for cancer therapy

**DOI:** 10.1186/s12929-019-0548-3

**Published:** 2019-07-17

**Authors:** Mohsen Keshavarz, Farid Solaymani-Mohammadi, Seyed Mohammad Miri, Amir Ghaemi

**Affiliations:** 1 The Persian Gulf Tropical Medicine Research Center, The Persian Gulf Biomedical Sciences Research Institute, Bushehr University of Medical Sciences, Bushehr, Iran; 2 Department of Virology, School of Medicine, Iran University of Medical Sciences, Tehran, Iran; 3 Department of Virology, School of Public Health, Tehran University of Medical Sciences, Tehran, Iran; 4 Department of Chemistry, Sharif University of Technology, Tehran, Iran; 5 Department of Virology, Pasteur Institute of Iran, P.O.Box: 1316943551, Tehran, Iran

**Correction to: J Biomed Sci (2019) 26:48**

**https://doi.org/10.1186/s12929-019-0542-9**

After publication of this article [1], it was brought to our attention that there are some errors in the section of ‘Authors’ contributions’.

The correct Authors’ contributions should be: MK, FS, and AG collected literature, designed and wrote the manuscript. FS and MM edited and prepared the manuscript for submission. All authors read and approved the final manuscript.
